# Mental Illness and Juvenile Offenders

**DOI:** 10.3390/ijerph13020228

**Published:** 2016-02-18

**Authors:** Lee A. Underwood, Aryssa Washington

**Affiliations:** 1 School of Psychology and Counseling, Regent University, Virginia Beach, VA 23464, USA; aryswas@mail.regent.edu; 2 Youth Development Institute, Phoenix, AZ 85006, USA

**Keywords:** juvenile justice, adolescent, mental illness, treatment programs

## Abstract

Within the past decade, reliance on the juvenile justice system to meet the needs of juvenile offenders with mental health concerns has increased. Due to this tendency, research has been conducted on the effectiveness of various intervention and treatment programs/approaches with varied success. Recent literature suggests that because of interrelated problems involved for youth in the juvenile justice system with mental health issues, a dynamic system of care that extends beyond mere treatment within the juvenile justice system is the most promising. The authors provide a brief overview of the extent to which delinquency and mental illness co-occur; why treatment for these individuals requires a system of care; intervention models; and the juvenile justice systems role in providing mental health services to delinquent youth. Current and future advancements and implications for practitioners are provided.

## 1. Introduction 

The juvenile justice (detention, probation, youth corrections facilities, *etc.*) system is currently faced with the task of providing mental health assessments and treatment services for its youth, as there is greater reliance on the juvenile justice system to do so. According to Garascia (2005), the juvenile justice system was originally both a rehabilitative and preventative approach, emphasizing the needs and rights of children over the appeal to punish them [1]. In accordance with The Juvenile Justice and Delinquency Prevention Act of 1974, the ultimate goal of juvenile justice was to divert youth from the formal, punitive processing of the adult justice system. This in turn resulted in the use of community-based programs rather than large institutions. The 1980s to the 1990s presented an interesting shift in the justice system’s treatment of juvenile offenders. Prior to the 1980s, juveniles were seen as rehabilitative; however, due to a short-lived surge in violent delinquency, protecting the community became the primary goal [2,3,4]. Consequently, the juvenile justice system developed an approach that uses a punishment/criminalization perspective over a rehabilitative/medicalization perspective [2,3,5]. Similar to the zero-tolerance attitude of the education system, in the early 1990s more than half of the states in the U.S. made revisions that allowed for juvenile offenders to be easily prosecuted in the adult criminal court and began to pass more punitive laws to address adolescent crime [2,6]. Although youth have committed violent and nonviolent crimes at a lower rate in the past few decades, Harms (2002) posits that the number of youth processed via the juvenile justice system has increased [7]. In 1960 approximately 1,100 delinquency cases were processed daily, while in 2009 juvenile courts handled about 4000 delinquency cases daily, and in 2013, approximately 2900 delinquency cases were processed daily [8]. The National Juvenile Justice Council (NJJC) estimates that the number of delinquency cases increased 30% between 1985 and 2009, however there was a 9% decrease between 1985 and 2013 [8]. More specifically, delinquency caseloads involving drug offenses, person offenses, and public order offenses increased, while property offense cases decreased between 1985 and 2013 [8]. The number of delinquency cases involving detention peaked in 2002, but decreased 44% through 2013 to the lowest level since 1985 [8]. According to the NJJC, despite the decrease in the volume of delinquency cases involving detention, the proportion of cases detained was larger in 2013 (21%) than in 1985 (19%).Between 1985 and 2013, the likelihood that a delinquency case would be handled informally (without petition for adjudication) decreased [8]. Although there was an intermediary increase, 31% of all delinquency cases resulted in either adjudication or waiver to criminal court in 2013, much similar to the 30% of all cases in 1985 [8]. It appears that some efforts have been made in the most recent years to decrease the number of youth cases processed in the juvenile justice system; however, this may be done by processing cases more informally or transferring cases to adult court. 

Greenwood (2008) posits that it would be more economically practical if the focus was placed on preventing juveniles from becoming adult criminals [9]. In recent years it has become more apparent that although incarceration and detainment is necessary for a small percent of juveniles, long-term confinement experiences tend to do more harm than good, often leading to continued offending and recidivism [10,11,12,13,14]. In contrast, community-based alternatives have been found to decrease re-offending, even for youth who commit serious and violent crimes [11,15]. During the 1990s, most states saw a reduction in the availability of public mental health services for children. Many communities began using the juvenile justice system to try to fill the gap caused by the decrease availability [11]. Additionally, public opinion regarding the US juvenile justice system has been shifting again from a punitive approach toward a rehabilitative model of care, mirroring the shift of the juvenile courts in recent years [10,11]. However, instead of focusing on community-based provision of services, an increased reliance on youth corrections systems to care for the mental health or other specialized needs of youth offenders has developed [11,16]. 

Trupin and Boesky (1999) note that as this shift occurred, many juvenile justice systems were left unequipped to deal with the acute needs of youth with mental health disorders [17]. Investigations by the United States Department of Justice (USDJ), have documented that the typically offered mental health services for youth in juvenile justice is often inadequate or unavailable [18].The Federal Advisory Committee on Juvenile Justice (2011) reports that barriers to providing adequate services include, insufficient resources, inadequate administrative capacity, lack of appropriate staffing, and lack of training for staff [19]. Due to the lack of research, inadequate models of care, insufficient policy development, ineffective experience and training of staff, and inadequate practice, juvenile correction personnel are quite hindered in being able to provide adequate services to youth offenders with mental health concerns.

To continue the shift toward juvenile offender rehabilitation, how systems of care intervene is of greatest import. There are generally four public systems that may respond when adolescents have problems affecting their welfare. These four systems concentrate in education, child protection, juvenile justice, and mental health [10,11]. Each of these systems has its own avenue or path for which an individual can gain entrance into the system—that is when the adolescent’s need fits the capabilities and objectives of the system. Recently, communities have begun to acknowledge that this model of separate service delivery does not consistently address the nature of adolescents’ needs [10,11]. Problems arise in effective treatment of adolescent offenders because many need services of more than one, if not all four, of the public systems of care at once. According to Grisso (2008), this is generally due to the fact that youths’ problems have interrelated causes and maintaining factors [11]. 

## 2. Mental Health Concerns for Youth in the Juvenile Justice System

The prevalence rate of youth with mental disorders within the juvenile justice system is found to be consistently higher than those within the general population of adolescents [20]. Estimates reveal that approximately 50 to 75 percent of the 2 million youth encountering the juvenile justice system meet criteria for a mental health disorder [6,16,21,22,23]. Approximately 40 to 80 percent of incarcerated juveniles have at least one diagnosable mental health disorder [16,24,25,26,27]. Two-thirds of males and three-quarters of females in previous studies of juvenile offender detention facilities, were found to meet criteria for at least one mental health disorder [26,28,29,30]. An additional one-tenth also met criteria for a substance use disorder [26,28,29,30].

Numerous comprehensive studies have indicated that there are certain types of mental disorders common among youth offenders, and that some of the symptoms increase youths risk of engaging in aggressive behaviors [16,26,31,32,33]. Additionally, risk of aggression is increased for many specific disorders and comorbid disorders because the emotional symptoms (*i.e*., anger) and self-regulatory symptoms (impulsivity) tend to increase the risk [10,16,26,31]. Commonly found mental health disorders in youth offenders include, affective disorders (major depression, persistent depression, and manic episodes), psychotic disorders, anxiety disorders (panic, separation anxiety, generalized anxiety, obsessive-compulsive disorder, and post-traumatic stress disorder), disruptive behavior disorders (conduct, oppositional defiant disorder, and attention-deficit hyperactivity disorder), and substance use disorders [11,30,34]. Of youth involved with the juvenile justice system, estimates suggest that approximately 15% to 30% have diagnoses of depression or dysthymia (pervasive depressive disorder) [35], 13% to 30% have diagnoses of attention-deficit/hyperactivity disorder, 3%–7% have diagnoses of bipolar disorder [16,36], and 11% to 32% have diagnoses of posttraumatic stress disorder [37]. Grisso (2008) also noted that both conduct disorder and substance use disorders are quite prevalent in youth in juvenile courts [11].

Heilbrun, Lee, and Cottle (2005) indicate that understanding the link between mental health difficulties and youthful offending is important in considering treatment response, as there is growing evidence that mental health difficulties are linked directly and indirectly to later offending behavior and delinquency [38]. Youth with mood disorders are more likely to display anger, irritability and hostility [39,40,41]. Mood disorders, mostly depression, occur in about 10%–25% of youth in the juvenile justice system [16,26,31]. The irritable mood that often accompanies depressive disorders increases youths’ probability of inciting angry responses from others, thereby increasing their risk of engaging in more physically aggressive acts that get them arrested [11,42,43]. In custody, the adolescent’s mood disorder may increase the risk of altercations with others or increase the risk of anger at oneself, resulting in self-injurious behaviors [11]. Typically, anxiety disorders in youth result in less aggressive behaviors with the exception of posttraumatic stress disorder (PTSD) [44]. Children and adolescents with PTSD are liable to respond to perceived threats aggressively and unexpectedly [44]. Psychotic disorders are rarely seen prior to early adulthood and rare in juvenile justice settings [11,32]. Nonetheless, some youth may display psychotic-like symptoms that are possible expressions of an early form of a psychotic disorder. However, Connor (2002) acknowledges that there is not much evidentiary support for claims that youth with evolving psychosis are a greater threat of aggression or harm than any other youth [32].

Grisso (2008) indicates that research has provided substantial evidence that youth with disruptive behavior disorders (conduct disorder, oppositional defiant disorder, and intermittent explosive disorder) display more physically aggressive behavior [10]. Additionally, the comorbidity of conduct disorder and attention-deficit/hyperactivity disorder (ADHD) has been linked to chronic and repeat offending during adolescence [45,46,47]. There is also substantial evidence for a relationship between substance use disorders and delinquency, as well as continued aggression into adulthood for substance abusing youth [28,48]. According to Angold and Costello (1993), co-morbidity, or the presence of more than one mental disorder, is common among adolescents with mental disorders [49], and approximately two-thirds of juvenile offenders meet the criteria for two or more disorders [45,46,47,50]. 

The high prevalence of mental disorders within the juvenile justice system does not necessitate a need for treatment, but emphasizes the need for different levels of mental health care with varying treatment options. Some youth who meet criteria for a disorder experience their disorder temporarily and only need emergency services. Others, approximately 10%, represent a group of youth with chronic mental health needs who will likely need clinical care well into adulthood [51]. Some youth function well despite their symptoms, while others present limited functionality. Regardless of the diagnosis, youth will present within the juvenile justice system differently, with different mental health needs requiring differing levels of care. This individuality requires an effective screening and assessment processes, as well as varied effective treatment options. This task is weighty for one system of care to provide fully.

## 3. Treatment Models

There is a multitude of evidence for the benefits of treating youth in acute distress due to mental illness. According to Grisso (2008), the most common and effective treatments include professional clinical care, psychopharmacology as needed, and the structuring of an environment to protect youth as well as reduce stress while in crisis [11]. Several types of psychotherapy and psychosocial interventions available for youth with mental disorders actually focus on youth with both mental health difficulties and delinquent behaviors. While evidence is limited for the efficacy and effectiveness of some approaches, there are a few specific therapeutic models with promising evidence for their effectiveness with youth offenders with mental disorders. 

### 3.1. Cognitive-Behavioral Interventions

Several studies have demonstrated that CBT is effective for reducing future delinquency for youth with various depressive and anxiety disorders [52,53,54]. Cognitive-Behavioral therapy (CBT) teaches youth awareness of social cues and promotes delaying, problem solving, and nonaggressive responding strategies. Cognitive-behavioral approaches are particularly effective with juvenile offenders. According to the National Mental Health Association (2004), this approach is quite effective for youth involved in the legal system as it is structured and focused on triggers of disruptive or aggressive behavior [55]. CBT has been used to address a variety of issues including interpersonal, problem solving, anger management, and social skills in individual or group treatment models [55]. Reductions in recidivism of up to 50 percent have been demonstrated in research studies [55]. Thinking For a Change (TFAC) is a cognitive behavioral intervention developed by Glick, Bush, and Taymans (2001). The program aims to restructure juvenile offenders’ thinking and teach pro-social cognitive skills by incorporating various cognitive approaches. Administered in a weekly small group for approximately two hours, the curriculum is comprised of 22 lessons focused on problem solving. Although evidence suggests that intensive cognitive behavioral skills training is quite helpful, Shelton (2005) found that programs that incorporate these treatment options are not the norm in most jurisdictions [54]. She purports that young offenders are often placed in programs modeled after those designed for adults. Another issue may be the adaptation of treatment interventions originally developed in outpatient or community settings, yet being used in secure or residential settings. While adapting treatment interventions for use in a different setting is common and often helpful, outcome data and research should be conducted to inform treatment effectiveness regarding the treatment’s intended use in the different setting. 

### 3.2. Integrated Co-Occurring Treatment Model

According to Cleminshaw, Sheppler, and Newman, the Integrated Co-occurring Treatment (ICT) model for youth is an integrated treatment program, and is a component model of care that uses treatment and service elements that are effective with similar populations but adapted to the specialized needs of youth with co-occurring mental health and substance abuse disorders [56]. It is currently utilized by a number of evidenced-based practices (*i.e*., Multisystemic Therapy, Multidimensional Family Therapy, and Functional Family Therapy). ICT uses a stage progression treatment approach (engagement, persuasion, active treatment, and relapse prevention) and engages motivational interviewing as a method to facilitate readiness for change [56]. Multisystemic therapy, Functional Family therapy, and Multidimensional Treatment Foster Care, are promising or effective treatments used for youth within the justice system [10,57,58]. These modalities incorporate aspects of treating juvenile offenders that Underwood and colleagues [59,60] have identified as beneficial and preventative when provided by the justice system. The following section provides an overview of programs being implemented in order to provide effective treatment for juvenile offenders with mental health concerns. 

### 3.3. Functional Family Therapy

Functional Family therapy (FFT), a brief family-centered approach, was developed in the 1960s in response to multi-need youth and families. Functional Family Therapy is often used for youth ages 11 to 18 at risk for and/or presenting with delinquency, violence, substance use, conduct disorder, oppositional defiant disorder, or disruptive behavior disorders [54]. One study found that youth receiving FFT had a 25 percent re-arrest rate, compared to a 45–70 percent re-arrest rate for those seen in juvenile court, or who had either no treatment or eclectic [54]. According to the national Mental Health Association (2004) a five-year follow-up study found that less than 10 percent of youth receiving FFT *versus* 60 percent of youth seen in juvenile court had subsequent arrests. While FFT has been shown to be an effective model for reducing recidivism, research also indicates that the training of behavioral health providers in the FFT model is essential [54].

### 3.4. Family Integrative Transition

The Family Integrative Transition (FIT) program combines empirically supported interventions such as, Multisystemic Therapy, Motivational Enhancement therapy (MET), Relapse Prevention, and Dialectical Behavior therapy (DBT). Aos (2004) described this rigorous treatment intervention as beginning two months prior to the juvenile’s release date and continuing for four to six months as the juvenile adjust to re-entry into the community [61]. The goal of FIT is to help youth generalize the skills learned while incarcerated to their daily lives within the community [62,63]. The FIT program is manualized, family-oriented, and community-based. The Juvenile Rehabilitation Administration (2002) indicates that the program was designed to address risk and protective factors of adjudicated youth with comorbid mental health and substance use disorders [62]. Evaluation research found that for those who participated in FIT, there was a 27 percent recidivism rate compared to 40 percent for non-participants [61].

### 3.5. Multisystemic Therapy

One of the best available treatment approaches for juvenile offenders with mental health treatment needs as indicated by empirical literature is Multisystemic Therapy (MST). An intensive, multi-modal, family-based approach, MST fits treatment with identified causal factors and correlating factors of delinquency and substance use [55]. Extant literature lends support for the effectiveness of MST with juveniles who have emotional and behavioral problems [55]. Studies have demonstrated reductions as high as 70 percent in rates of re-arrest, reductions in out-of-home placements up to 64 percent, improvements in familial functioning, and decreases in mental health concerns for serious juvenile offenders [55].Timmons-Mitchell *et al.*, (2006), found that that the use of MST produced significant reductions in rearrests and improvements in four areas of functioning measured by the Child and Adolescent Functional Assessment scale at 18 months and 6 months’ post treatment [64].This study used a real-world mental health setting with juvenile justice involved youth, further supporting the claim that community-based treatment may best fit the needs of delinquent youth with mental health difficulties. A meta-analysis of MST outcome studies [65] found that effect sizes of MST efficacy studies tend to be quite larger than MST effectiveness studies [66,67,68]. 

### 3.6. Wraparound Approach

Burns and Goldman (1999) define wraparound as a “philosophy of care that includes a definable planning process involving the child and family that results in a unique set of community services and natural supports individualized for that child and family to achieve a positive set of outcomes” ([69], p. 10). This framework lends better treatment support for the notion that youth with complex emotional or behavioral problems are often involved in more than one system of care. Wraparound services link the youth’s strengths and needs to services and supports within his or her community. The wraparound process is related to the system-of-care framework. Generated by the Child and Adolescent Service System Program (CASSP), Systems-of care are comprehensive programs that use a coordinated network of mental health and other support services to meet the evolving needs of children and adolescents with severe emotional problems [69]. Research shows that the wraparound process is challenging, yet promising in treating the mental and emotional needs of youth in the justice system. The Wraparound Milwaukee program is excelling in its collaborative efforts. The program has successfully integrated juvenile justice, mental health, child welfare, and education systems to provide services to youth. Additionally, each youth receives an individually tailored treatment plans. Outcome evaluations revealed a 60 percent reduction in the use of residential treatment and an 80 percent decrease in psychiatric hospitalization [70]. Suter and Bruns (2009) meta-analysis examining the effectiveness of wraparound processes revealed quite a few gaps in research that limit the capacity to claim wraparound services as an effective treatment despite its promise [71]. As they included only experimental and quasi-experimental designs, there were only seven controlled outcome studies.The researchers found that effect sizes were positive and significant, but small when examining specific outcomes. Juvenile-justice related outcomes (not defined) was also significant but small in effect size [72]. Essentially, the results indicate that there is a real difference between those receiving wraparound care *versus* those who are not; however, the magnitude of these differences is quite small when studying specific outcomes.This finding indicates the necessity for careful comparison of treatment services with a larger sample size, and very specific and valid definitions and measures of outcomes. While wraparound programs could make positive impacts, Suter and Brun [71] caution that research studies have been limited by study designs, comparability among groups, and unreported levels of attrition. As such, it does not meet the criteria of an evidence-based treatment as of yet [71].

### 3.7. Multidimensional Treatment Foster Care

Multidimensional Treatment Foster Care (MTFC) is an alternative to group, residential, secure-care, or hospitalization treatment for adolescents with severe and chronic emotional and behavioral disorders [54]. Adolescents are placed with trained, local, and supervised families for approximately six to nine months. Throughout the MTFC placement, family therapy is also conducted. According to the National Mental Health Association (2004), outcome research regarding MTFC programs has demonstrated that youth spent 60 percent fewer days incarcerated than those not receiving services, and also had significantly fewer arrests [54]. Chamberlain *et al.* (2007) and Leve, Chamberlain, and Reid (2005) found that results from prior studies of girls support the efficacy of MTFC relative to services as usual (group care intervention) on targeted outcomes such as, criminal referrals, days in locked setting, self-reported delinquency, and deviant peer affiliation [71,72]. MTFC also proved efficacious for non-targeted outcomes such as, pregnancy, school attendance, and completion of homework [73,74]. Harold and colleagues (2013) found the MTFC decelerated girl’s depressive symptoms and showed greater benefits for girls with higher levels of initial depressive symptoms [75,76].

### 3.8. Crisis Intervention Teams

Doulas and Lurigio (2010) discussed one of the newest, specialized law enforcement programs in the US—Crisis Intervention Teams (CITs) for youth with mental illness [77]. The development of J-CITs (juvenile-crisis intervention teams) was a response to the fragmented and often inadequate behavioral health services for youth across the educational, juvenile justice, and mental health systems [77]. While the number of adolescents diagnosed with mental illness has risen in the United States, a large amount of youth are never diagnosed or never treated [77]. Specifically, CIT programs were developed by communities in order to address the school to prison pipeline, allowing for the diversion and referral of adolescents with mental disorders for services. Doulas and Lurigio (2010) examined three programs in Denver, Chicago, and San Antonio in the early stages of implementation. Initial findings indicate the need and utility of such programs [77] Children in Crisis (CIC) Denver, implemented by the police department in 2010, aims to recognize mental illness, and offer resources to provide follow-up care for youth in distress. Police first deescalate the crisis and then refer the youth for services. CIC training includes information on trauma and adolescence, how to approach traumatized youths, developmental milestones, common mental illnesses among adolescents, response tactics during calls, and the nature of psychiatric emergencies [77]. Officers are also assisted in how to interact with youth with developmental disabilities via a role-playing component. Studies evaluating the CIC process and outcomes are necessary [77].

The most effective treatment models that have demonstrated delinquency-reducing benefits for youth with mental disorders include Functional Family Therapy, Treatment Foster Care, and Multisystemic therapy. Interestingly, all of these therapeutic models are similar in that they involve families and youth, are community-based, and deal with problem behaviors and stresses as a systemic family unit. Essentially these treatment models represent an integrated system of care. Grisso (2008) noted the aforementioned interventions are the few that have demonstrably reduced recidivism of youth with mental disorders [11]. Research regarding each of the mentioned models is lacking with regards to efficacy and effectiveness, as many of the studies reveal problems with study design, small effect sizes, and other confounding variables. However, the greatest issue related to treating juvenile offenders with mental disorders does not appear to be limited evidence-based or effective treatments, as much as how and where these treatment models should be provided in order to be most efficacious. Cuellar, Markowitz and Libby (2004) found that youth in foster care who received community-based services had lower subsequent rates of pretrial detention center admissions [78]. Additionally, adjudicated youth with mental disorders who were diverted from institutional placement and received services in the community had significantly fewer arrests than similar youth who received no treatment, according to Cuellar, McReynolds, and Wasserman (2005) [79].

## 4. Response to Treatment Needs

Responses to the needs of youth with mental disorders in the juvenile justice system often focuses on generating more treatment services within the juvenile justice system [11]. Grisso (2008) suggests that these youths would benefit more by defining what is meant by treatment and by avoiding dependence on the juvenile justice system to respond to broad issues such as adolescent mental health and crime [11]. Current reasoning and research posits that the role of the juvenile justice system should be concentrated, narrow, and based on collaboration with the broader community to meet the needs of youth with mental health disorders [11]. To a certain degree it makes sense that the juvenile justice system would be where society focuses efforts to treat delinquent youth with mental disorders; however, this practice can be detrimental to the youth and create an economic strain on funding within the juvenile justice system.

Putting so much of the community’s limited mental health resources into juvenile justice programs generates the opportunity to criminalize youth with mental health difficulties, or place youth in the most restrictive form of care in order to get them the best resources. Consequently, if funding for children’s mental health services are limited and allocated to the juvenile justice system, then the community’s ability to develop varied community-based services is limited. As a result, and as has been seen historically in the juvenile justice system, when community-based services are reduced, more youth are referred to the juvenile justice system [80]. Tonry and Moore (1998) posit that when youth must be placed in more restrictive settings in order to receive basic mental health services, the likelihood of future delinquency increases, as does criminal behavior and arrests as adults [81].

Legal considerations restrict treatment options for youth arrested and detained. Pretrial detention centers are required to provide emergency mental health services for youth in crises; however, the juvenile justice system cannot impose rehabilitative or longer-range mental health interventions until a youth is adjudicated, or comes under the custody of the juvenile justice system. Clinical considerations also suggest that the juvenile justice system may not be able to adequately treat all delinquent youth with mental health needs. Grisso (2008) posits that it is possible that some delinquent youth with mental disorders might be rehabilitated within the structure and guidance of properly operated, secure-care facilities, but trust and caring, essential components of a therapeutic relationship, are difficult to maintain when the therapist is viewed as part of the system that restricts youth’s liberty [11]. In fact, some treatments performed in secure care facilities can be counterproductive. Group therapies involving youth exhibiting many antisocial behaviors sometimes have negative effects on peers with less antisocial behaviors [82]. Additionally, Lipsey and Wilson (1998) suggest that considerable evidence indicates that rehabilitation methods in secure settings like behavior modification effectively change behavior within the setting, but the skills do not transfer well to the community setting of the youth [83]. 

Recently, how to best respond to delinquent youth with mental disorders has begun to focus on a community system of care that integrates services across child mental health, child protection, education, and juvenile justice agencies. Many youths have multiple needs that require services from more than one agency. Although a youth may receive services from various agencies, there is often a lack of coordination between the systems of care that creates conflict, inefficiency, frustration, and sometimes harm. A community system of care seeks to improve cross-agency referral and collaboration, and possibly even cost-sharing for the development of uncommon services [11,84,85]. Nationwide, many communities have generated and employed community systems of care. In these systems, treatment of juvenile offenders with mental health needs is the responsibility of each agency of care, not merely the juvenile justice system. Grisso (2008) concludes that this collaboration allows for the juvenile justice system to divert many youths from entering detention centers with the ability and capacity to refer them to community programs and to develop better aftercare plans for youth reintegrating into the community [11]. Duchnowski, Kutash, and Friedman (2002) found that initial research has documented benefits of community systems of care with regard to both economic and child welfare outcomes, as well as reductions in recidivism [86]. As the mental health needs of delinquent youth become the collective responsibility of the community, then the role that the juvenile justice system plays must be redefined.

## 5. The Role of the Juvenile Justice System

Grisso (2004) posits that the role of the juvenile justice system would still be considerable, but more focused and limited than if it were the sole provider of mental health services for juvenile offenders [87]. Also, the primary role of the juvenile justice system would vary at different stages in processing youth offenders. The first stage is the youth’s arrest and referral to juvenile court. At this stage the primary role should be to identify youth with mental disorders who can be diverted from processing to the community where treatment services are based rather than remaining in pretrial detention or proceeding to full juvenile justice processing [11,60]. This diversion is readily feasible with youth referred to detention centers for minor offenses or those who present with no danger to others. Many youths with mental health needs could be diverted from formal juvenile justice processing if their mental health problems were identified at this early stage and if policies and system-of-care options (foster and shelter care services) were in place. 

During stage two, the pretrial detention, the juvenile justice systems should maintain the emergency service provision obligation for youth awaiting trial, however, this should generally be the extent of the juvenile justice systems role. All detention centers should have the capacity to respond to mental health emergencies (*i.e*., suicide risk, symptom escalation), but do not necessarily need to have mental health professionals always on staff. This would require facilities to have clear staff procedures for responding to youth emergency needs, access to clinical consultants, and arrangements for rapid transfer to psychiatric facilities, according to Grisso (2008) [11]. The procedure may look similar to the aforementioned crisis intervention teams (CITs). Despite the high prevalence of mental disorders in pretrial detention centers, approximately 25% to 30% of detained youth with mental disorders actually receive treatment while in detention [88]. Much more research is required to determine the level of need in detention centers based on symptom levels of youths’ mental disorders as opposed to merely diagnosis. 

According to Grisso (2008), stage three is the assessment for dispositional treatment planning stage [11]. When youth are adjudicated, the courts tend determine the appropriate placement for rehabilitation. Screening at this time also requires identifying mental health needs, however, the purpose is to specify types of longer-term mental health treatments for their rehabilitation plan. Assessment at this stage should help identify youth with mental disorders who, despite being adjudicated, might benefit from rehabilitation in non-secure community placements where they might benefit from a variety of mental health services typically unavailable in secure-care [11,89]. 

Grisso (2008) suggests that stage four of processing in the juvenile justice system is for youth placed in secure care or transitioning out of a secure facility into the community [11]. The juvenile justice system can meet the mental health needs of youth in secure care by buying psychiatric consultation services or by hiring mental health professionals to provide psychosocial interventions. For the small percent of youth with serious, chronic, and persistent mental disorders too disturbed to function within the structure of most youth secure-care programs, specialized “clinical units” are sometimes developed. On these units, youth with serious, disruptive mental disorders may be separated from the general youth correctional population and or receive specialized clinical services from fulltime mental health professionals. Ideally, a model that blends the resources of the juvenile justice system and the child mental health system to operate and staff such facilities would be most advantageous. Grisso (2008) acknowledged that such facilities exist in some states, but they have not been “modeled” or studied in a way that would allow for their systematic development nationwide [11]. New issues may arise when youth are released from secure care back into the community.

Across the United States, several states have generated and implemented programs within their juvenile justice system structures that address the mental health concerns of youth offenders. Many of these programs implement some aspects of the aforementioned recommendations presented by Underwood and colleagues [59,60]. Arizona, California, Colorado, and New Hampshire have all established courtroom procedures enabling legal personnel to request mental health screenings for juveniles involved in delinquency proceedings, while other jurisdictions have created specialized courts to serve youth with mental health needs [70]. Some states have community-based treatment programs for juveniles that do not pose a danger to public safety and for whom detention may exacerbate their psychological disorder [70]. Additionally, assessment with diversion at the early stage in the juvenile justice process is a promising prevention intervention [70]. Diversion programs have been implemented in many jurisdictions so that juveniles may complete certain requirements as opposed to being processed for adjudication [70]. An important part of a comprehensive approach entails providing juveniles with access to mental health services after being released from secure care facilities [59,70]. Legislation in Virginia and Texas requires Juvenile Justice to establish regulations for continuity of care regarding mental health, substance use, and other therapeutic treatments for youth re-entering the community post-commitment or detainment [70].

## 6. Current and Future Advancements

According to Grisso (2008), compared to youth without mental disorders, youth with mental disorders commit only a minority of a community’s delinquencies, but they have a greater risk of offending and re-offending than youth on average [11]. A great deal more research is necessary in order to speak confidently about the best policies for responding to the mental health needs of youth offenders; however, certain directions for appropriate policies are evident. The shift of the juvenile justice system as whole towards a more rehabilitative *versus* punitive model of care appears to be in the right direction. The role of the juvenile justice system in meeting the mental health needs of youth offenders must become more focused and limited, yet collaborative with the child protection, education, and child welfare agencies. Instead of focusing on generating more evidence-based treatments to be used within the juvenile justice system, research seems to suggest that diversion programs and more community-based treatment services would be most beneficial to youth delinquents with mental health difficulties. In order to develop and implement such services; a very clear and standardized screening and assessment process is required. Evidence-based screening and assessment tools should be used universally at the aforementioned decision points in juvenile processing to identify youth with mental health needs. Additionally, every juvenile justice intake (assessment center) and detention program should document and archive screening and assessment results to provide data needed for system planning and resource development, especially for those specific to the communities from which youth come. Also, it seems that prioritizing educating personnel about mental health problems of youth, will also likely improve the system’s ability to identify and respond appropriately to such needs. Because of the multiple needs that delinquent youth with mental disorders present with, all policies should be united by an overarching approach that reduces the political distance and boundaries among existing child welfare systems.

## 7. Conclusions

In recent years it has become apparent that incarceration and detainment, while necessary for a small portion of juveniles, tends to have more detrimental effects including continued offending and recidivism. From an economical and long-term benefit standpoint, community-based alternatives have been found to be more successful with rehabilitating youth, even for youth who commit serious and violent crimes. To this end, an integrated system of care (education, child protection, juvenile justice, and mental health) must intervene in juvenile cases in a collaborative manner in order to meet the interrelated needs of each individual youth. Diagnoses aside, youth present within the juvenile justice system, requiring different levels of care. As such, rehabilitation requires an effective screening and assessment process with varied treatment options. CBT, ICT, FFT, FIT, MST, MTFC, and Wraparound treatment models are identified as effective treatment models for juvenile offenders. The models of treatment are most effective when they involve, thoroughly trained professionals, families and youth, are community-based, and deal with problem behaviors and stresses as a systemic unit. Research indicates that the mental health needs of delinquent youth must become the collective responsibility of the community, thus requiring a redefinition of role played by the juvenile justice system. This role should be concentrated, narrow, and based on collaboration with the broader community to meet the needs of offending youth with mental health disorders. The use of Juvenile Crisis Intervention Teams in some states is an initial step in diverting and referring youth offenders to resources within the community. The initial role of the juvenile justice system should be in identifying mental health needs and diverting youth to the community. At different points throughout the processing of juvenile offenders, the juvenile justice systems role should include assessment with the purpose of identifying needs and formulating rehabilitation plans that include varied treatment options. For youth placed in secure-care or for youth transitioning to the community, most effective models of treatment will include psychosocial interventions carried out by mental health professionals and an after-care plan with services to help the youth offender transfer and maintain learned skills. As opposed to focusing resources on creating new interventions within the juvenile justice system, the literature indicates that redefining the roles of the juvenile justice, education, mental health, and child protection systems to be a systematic and collaborative unit of care will be more effective in rehabilitating youth offenders. 
